# Efficacy of new-generation antidepressants assessed with the Montgomery-Asberg Depression Rating Scale, the gold standard clinician rating scale: A meta-analysis of randomised placebo-controlled trials

**DOI:** 10.1371/journal.pone.0229381

**Published:** 2020-02-26

**Authors:** Michael P. Hengartner, Janus C. Jakobsen, Anders Sørensen, Martin Plöderl

**Affiliations:** 1 Department of Applied Psychology, Zurich University of Applied Sciences, Zurich, Switzerland; 2 The Copenhagen Trial Unit, Centre for Clinical Intervention Research, Rigshospitalet, Copenhagen University Hospital, Copenhagen, Denmark; 3 Department of Regional Health Research, The Faculty of Heath Sciences, University of Southern Denmark, Odense, Denmark; 4 Department of Cardiology, Holbæk Hospital, Holbæk, Denmark; 5 Nordic Cochrane Centre, Rigshospitalet, Copenhagen, Denmark; 6 Department of Crisis Intervention and Suicide Prevention, Christian Doppler Klinik, Paracelsus Medical University, Salzburg, Austria; Chiba Daigaku, JAPAN

## Abstract

**Background:**

It has been claimed that efficacy estimates based on the Hamilton Depression Rating-Scale (HDRS) underestimate antidepressants true treatment effects due to the instrument’s poor psychometric properties. The aim of this study is to compare efficacy estimates based on the HDRS with the gold standard procedure, the Montgomery-Asberg Depression Rating-Scale (MADRS).

**Methods and findings:**

We conducted a meta-analysis based on the comprehensive dataset of acute antidepressant trials provided by Cipriani et al. We included all placebo-controlled trials that reported continuous outcomes based on either the HDRS 17-item version or the MADRS. We computed standardised mean difference effect size estimates and raw score drug-placebo differences to evaluate thresholds for clinician-rated minimal improvements (clinical significance). We selected 109 trials (n = 32,399) that assessed the HDRS-17 and 28 trials (n = 11,705) that assessed the MADRS. The summary estimate (effect size) for the HDRS-17 was 0.27 (0.23 to 0.30) compared to 0.30 (0.22 to 0.38) for the MADRS. The effect size difference between HDRS-17 and MADRS was thus only 0.03 and not statistically significant according to both subgroup analysis (p = 0.47) and meta-regression (p = 0.44). Drug-placebo raw score difference was 2.07 (1.76 to 2.37) points on the HDRS-17 (threshold for minimal improvement: 7 points according to clinician-rating and 4 points according to patient-rating) and 2.99 (2.24 to 3.74) points on the MADRS (threshold for minimal improvement: 8 points according to clinician-rating and 5 points according to patient-rating).

**Conclusions:**

Overall there was no meaningful difference between the HDRS-17 and the MADRS. These findings suggest that previous meta-analyses that were mostly based on the HDRS did not underestimate the drugs’ true treatment effect as assessed with MADRS, the preferred outcome rating scale. Moreover, the drug-placebo differences in raw scores suggest that treatment effects are indeed marginally small and with questionable importance for the average patient.

## Introduction

The debate whether antidepressants are an effective treatment for depression is ongoing and unresolved [1–5]. Although meta-analyses unequivocally produce statistically significant drug-placebo differences in acute treatment trials [6–8], various researchers showed that these differences are so small that their practical relevance is questionable [9–12]. A common reply to these critics is that the most common outcome measure in depression trials, the Hamilton Depression Rating Scale (HDRS), has poor validity, is not unidimensional, and is not sensitive to symptom change because it contains items that presumably capture adverse effects of antidepressants rather than core depression symptoms [13–15]. According to this view, the wide-spread application of the HDRS has resulted in a significant underestimation of antidepressants’ true treatment effects.

An alternative approach to examine the efficacy of antidepressants would be to base effect size estimates on an outcome that is widely accepted as a reliable and valid measure of depression. One such outcome is the Montgomery-Asberg Depression-Rating Scale (MADRS), which was constructed to be particularly sensitive to change and to treatment effects on core depression symptoms [16]. Independent evaluations have confirmed that the MADRS is psychometrically superior to the HDRS, that it is unidimensional, and that it should be the preferred outcome measure [17]. In accordance, the MADRS is considered the “gold standard clinician rating scale for depression” [18].

The aim of this meta-analysis was thus to re-evaluate the data from short-term antidepressant trials for adults with major depression collected by Cipriani et al. [6] by focusing on differences in effect size estimates for MADRS and HDRS. This comparison will empirically test the claim that the predominant use of HDRS has resulted in an underestimation of antidepressants’ true treatment effects. If this assumption was true, then effect size estimates for the MADRS should be substantially larger than estimates based on the HDRS. Given that the interpretation of effect sizes is not straightforward (e.g. does an effect size of 0.3 represent a practically relevant effect [9]?), we will further examine drug-placebo differences in raw scores for both rating scales to evaluate the clinical significance of the drugs’ average treatment effect. If antidepressants provide clinically significant treatment effects on core depression symptoms, then the drug-placebo difference in MADRS raw scores should exceed the threshold of a predefined minimal important difference.

## Methods

Since this a post-hoc analysis of a freely available dataset, we did not write a study protocol and did not pre-register the planned analysis. That is, we did not conduct our own literature search, but relied on the work by Cipriani et al. [6]. Except for this omission, the study was conducted and reported according to the PRISMA statement [19] and used established procedures detailed in the Cochrane handbook [20].

### Data source, study selection and outcomes

**Fig 1:**
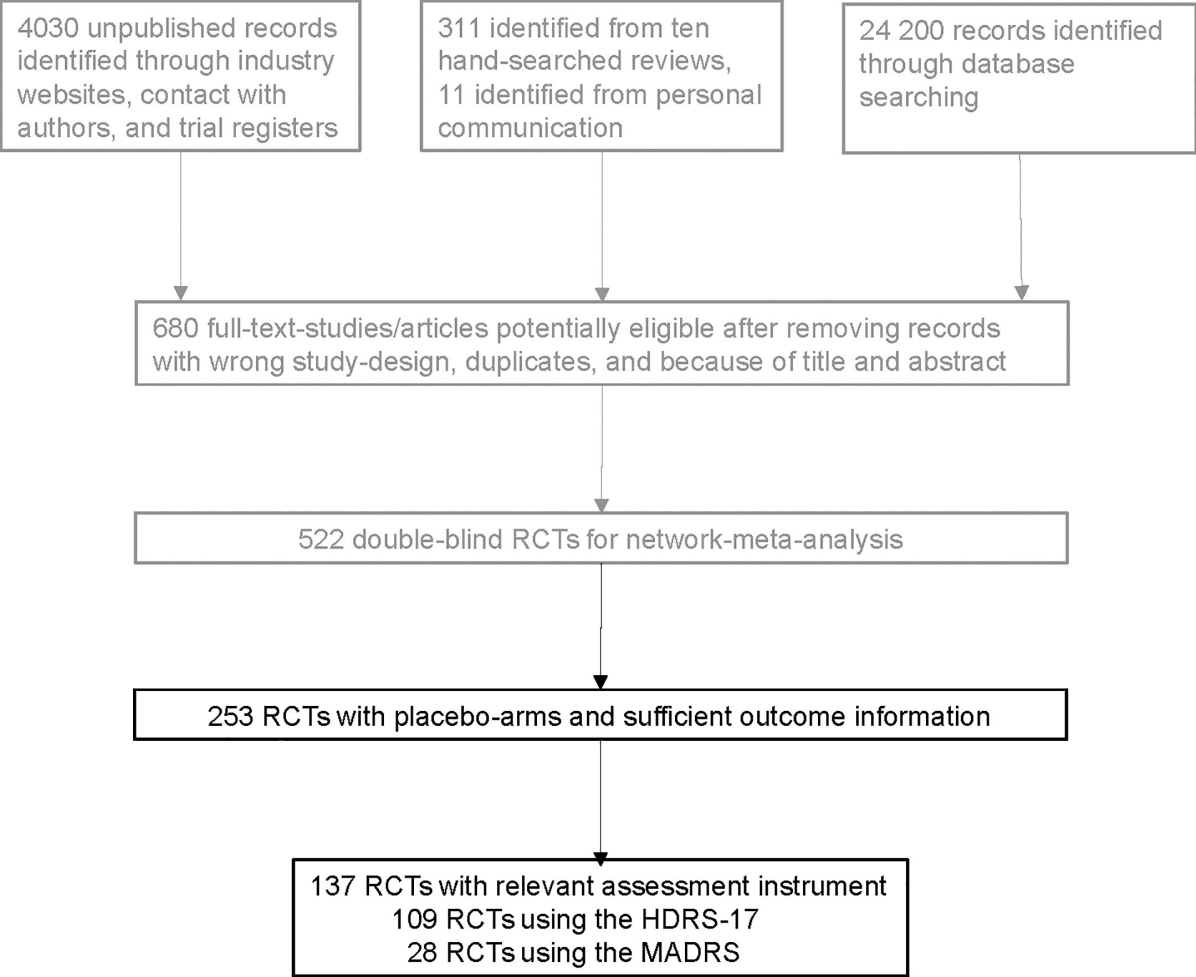
PRISMA study flowchart. Literature search and study selection conducted by Cipriani et al. [6] is depicted in grey, our own further study selection for this particular analysis is depicted in black. RCTs: randomized controlled trials; HDRS-17: Hamilton Depression Rating Scale 17-item version, MADRS: Montgomery-Asberg Depression Rating Scale.

Our analysis was based on short-term, mostly 8-week clinical trials of antidepressants for adults with unipolar major depression, reported in a recent systematic review by Cipriani et al. [6]. The authors of this comprehensive study made the data available in a public repository (https://data.mendeley.com/datasets/83rthbp8ys/2). This is the largest database of new-generation antidepressants for the acute treatment of major depression compiled thus far. The comprehensive literature search conducted by Cipriani et al. [6] yielded 522 trials (with 21 individual antidepressants), of which we extracted 253 trials owing to the following inclusion criteria: first, they included a placebo-arm and, second, they also reported any outcome measure. However, various trials reported only response rates or used continuous outcome scores other than ratings based on either HDRS-17 or MADRS. Therefore, of the 253 eligible trials, we selected 109 (43%) that reported continuous outcomes based on the HDRS-17 and 28 (11%) based on the MADRS. The study flowchart is shown in Fig 1.

When trials had multiple treatment arms with different doses of antidepressants, these arms were aggregated. This is an appropriate procedure, as low-to-medium doses achieve the highest efficacy [21]. When trials compared different antidepressants, the data of these arms were aggregated to have only one estimate for the overall analysis of the active drug relative to placebo. The selected trials either reported pre-post differences (n = 169, 67%) or drug-placebo differences at the end of treatment (n = 84, 33%). Since results for both outcomes were similar, we did not report the analysis separately, as in previous publications with the same dataset [6, 11]. In addition to HDRS-17 and MADRS scores we also extracted year of publication, drop-out-rate, sample size, and publication status (published vs. unpublished) from the database. We did not conduct a formal risk of bias assessment, as this was extensively studied by Munkholm et al. [11] in a previous analysis of this dataset.

### Statistical analysis

We calculated the standardised mean differences for each trial and aggregated them by means of a random effect meta-analysis based on the inverse variance method according to the procedure suggested by Munkholm et al. [11] using the metafor package in R (version 3.4.3). We ran a similar analysis using the raw score mean-differences to evaluate the clinical significance of the summary estimates. For the analysis of individual antidepressants, only comparisons with at least 3 trials using either the HDRS-17 or the MADRS were selected.

We used subgroup analysis and meta-regression to test for differences between the HDRS and MADRS. Effect size estimates with corresponding 95% confidence intervals (CI) were based on Hedges’ g standardised mean difference method and the variability of effect sizes due to heterogeneity was calculated as I^2^. Test statistics and confidence intervals were adjusted according to the procedure by Hartung and Knapp, as recommended in the Cochrane handbook [20].

Classifying effect sizes as small or large based on arbitrary thresholds is not recommended [22]. We therefore proposed that a more stringent approach to the evaluation of antidepressant efficacy would be to put the drug-placebo difference in raw scores into context and to compare them to empirically derived cut-off scores for clinical significance [9]. As demonstrated by Leucht and colleagues, for a clinician to detect a minimal improvement at least 7 points are required on the HDRS-17 [23] and about 8 points on the MADRS [24]. Moreover, with respect to the minimal important difference in patient-oriented outcomes (mostly quality of life), Barrett et al. [25] detail that across various health problems, scores of about 7–9% of the maximum scale score are necessary. With respect to the HDRS-17 (maximum score 52), the minimal important difference would thus be about 4 points, while for the MADRS (maximum score 60), it would be about 5 points. If the upper bound of the confidence interval for the effect size estimate is smaller than those thresholds, we consider that antidepressants’ average treatment effects are clinically not significant.

A sensitivity analysis included a meta-regression across all antidepressants and controlled for potential confounders that differed between trials using the HDRS-17 and the MADRS. These covariates included year of publication, drop-out-rate, sample size, and publication status. We also cross-validated our findings with the standard methods for continuous outcomes proposed in the Cochrane handbook, the DerSimonian-Laird random-effects meta-analysis and the inverse-variance fixed-effects method [20].

## Results

### Differences between HDRS and MADRS

For the overall analysis across different antidepressants, there were 137 trials/comparisons. A full list of included studies is provided in the online supplement (S1 File) and for a description of these studies see Cipriani et al. [6]. For the HDRS-17, the trials included a total of 32,399 patients, of which 19,796 received the active drug and 12,603 received placebo. For the MADRS, a total of 11,705 patients were included; 7477 received active drug and 4228 received placebo. As detailed by Munkholm et al. [11], most studies were rated at high or unclear risk of bias with respect to allocation concealment and blinding of participants.

Aggregated across all drugs, the summary effects size (ES) for the HDRS-17 and the MADRS were almost identical (ES = 0.27, CI = 0.23–0.30 vs. ES = 0.30, CI = 0.22–0.38), and the difference was statistically not significant based on both subgroup analysis (Q = 0.53, df = 1, p = 0.47) and meta-regression (b = -0.03, SE = 0.04, p = 0.44). Observed variability across studies, which could indicate between-study heterogeneity, was low for the HAMD-17 (I^2^ = 45%) and moderate for the MADRS (I^2^ = 74%). The forest plot for the meta-analysis across all drugs is shown in the online supplement (S1 File).

**Fig 2:**
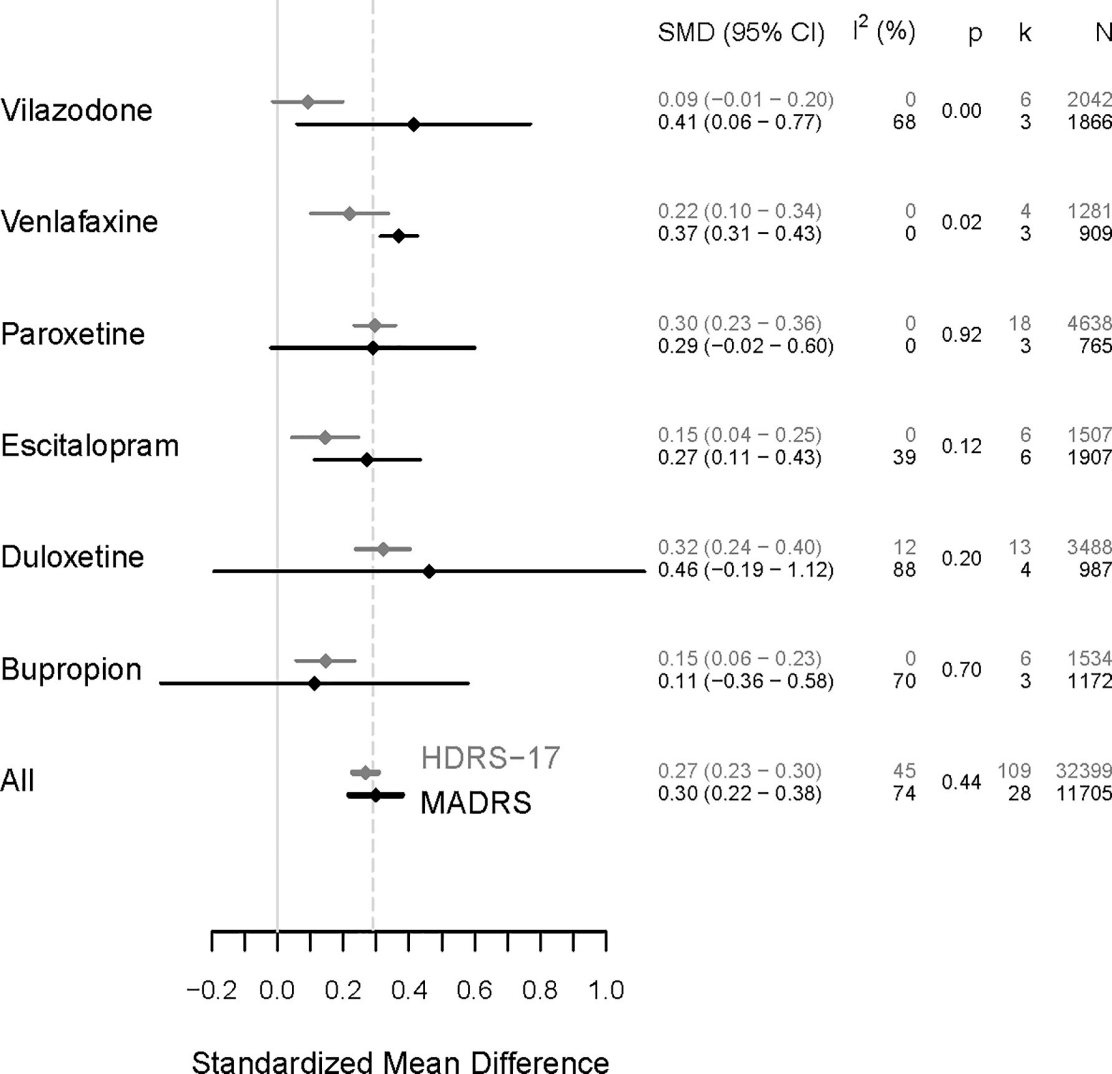
Meta-analytic results across all antidepressants and for individual antidepressants with sufficient number of trials (k≥3). Horizontal lines denote the 95% confidence intervals of the point estimates. Meta-analytic results for trials using the HDRS-17 and the MADRS are depicted in grey and black, respectively. The vertical dashed line indicates the overall result (SMD = 0.29) from the Cipriani et al. [6] dataset as reported by Munkholm et al. [11].

Analysis of individual drugs (k≥3 for both HDRS-17 and MADRS) was possible for bupropion (k = 9), duloxetine (k = 17), escitalopram (k = 12), paroxetine (k = 21), venlafaxine (k = 7), and vilazodone (k = 7). The results for the individual drugs are shown in Fig 2. Although some effect size estimates differed between HDRS-17 and MADRS, confidence intervals overlapped for all individual drugs. In the meta-regression analysis, the differences achieved statistical significance for venlafaxine (b = -0.15, SE = 0.05, p = 0.02) and vilazodone (b = -0.32, SE = 0.08, p = 0.005), but not for paroxetine (b = 0.01, SE = 0.08, p = 0.92), escitalopram (b = -0.13, SE = 0.08, p = 0.12), duloxetine (b = -0.17, SE = 0.12, p = 0.20), and bupropion (b = 04, SE = 0.09, p = 0.70). After correction for multiple testing (α = 0.008), the effect size comparison between HDRS-17 and MADRS remained statistically significant only for vilazodone. The statistical tests based on subgroup analysis were nearly identical to the results of the meta-regression and thus are not reported here. Forest plots for all individual drugs are shown in the online supplement (S1 File).

SMD (95% CI): standardized mean difference based on Hedges’ g and corresponding 95% confidence interval, I^2^: heterogeneity index, p: p-value of the meta-regression analysis with the instrument (HDRS-17 vs. MADRS) as predictor variable, k: number of trials, N: number of participants (antidepressant plus placebo arm).

### Clinical significance of treatment effects

As detailed in the methods section, the pre-defined threshold for a minimal improvement according to clinician evaluation is 7 points on the HDRS and 8 points on the MADRS. Corresponding cut-offs for a minimal important difference according to patient ratings are about 4 points for the HDRS-17 and 5 points for the MADRS. In the present meta-analysis, the drug-placebo difference in raw scores was 2.07 (CI = 1.76–2.37) for the HDRS-17 (possible range 0–52 points) and 2.99 (CI = 2.24–3.74) for the MADRS (possible range 0–60 points) (S1 File). Drug-placebo differences, for both HDRS-17 and MADRS, were thus clinically not significant. The drug-placebo difference of 2.07 points on the HDRS-17 corresponds to 4% of the maximum scale score (52 points), whereas the difference of 2.99 points on the MADRS corresponds to 5% of the maximum scale score (60 points). These almost identical figures again demonstrate that the drug effects assessed with HDRS-17 and MADRS do not differ meaningfully.

### Sensitivity analysis

In univariate meta-regression analysis, trials using the HDRS-17 and the MADRS differed by year of publication, dropout rate, and sample size, but not by publication status. In a multivariable meta-regression analysis that included all significant confounding variables as additional predictor variables, the overall effect of the instrument (HDRS-17 compared to MADRS) was statistically not significant and very small (B = -0.07, SE = 0.04, p = 0.06). The results were almost identical when different meta-analytic methods were used. The inverse-variance fixed-effects model produced estimates of ES = 0.25 (0.23–0.28) and ES = 0.30 (0.27–0.34) for HDRS-17 and MADRS, whereas based on the DerSimonian-Laird random-effects model the estimates were ES = 0.26 (0.23–0.29) and ES = 0.30 (0.22–0.38).

## Discussion

### Principal findings

Our meta-analysis found a mean effect size of 0.30 (0.22–0.38) for the MADRS as compared to 0.27 (0.23–0.30) for the HAMD-17. That is, the effect sizes for both rating scales are comparable when averaged over all drugs. Although the MADRS is considered the gold standard [18] and the preferred depression rating scale due to its good psychometric properties [17], it does, overall, not produce higher effect size estimates than the HDRS-17, which is considered “psychometrically and conceptually flawed” [26]. Therefore, contrary to prominent claims, there is no compelling evidence to assume that the efficacy of new-generation antidepressants is underestimated due to the wide-spread application of the “unresponsive” full HDRS scale [14, 15, 27]. Moreover, with an estimated 2.07 drug-placebo difference in raw scores for the HDRS-17 (4% of the maximum scale score) and a 2.99 difference for the MADRS (5% of the maximum scale score), the drugs’ treatment effect falls well below the empirically-derived thresholds that both clinicians and patients would consider a minimal improvement [23–25]. Across various health problems, scores of about 7–9% of the maximum scale score are necessary for patients to notice a minimal important difference [25], whereas for a clinician to detect a minimal improvement at least 7 points are required on the HDRS-17 [23] and about 8 points on the MADRS [24].

We cannot preclude with certainty that there is no difference between HDRS-17 and MADRS for all individual drugs. After correction for multiple testing, for vilazodone the effect size based on MADRS was statistically significantly larger than the effect size produced by the HDRS-17. However, it is important to note that vilazodone’s HDRS-17 effect size (ES = 0.09) was considerably lower than the summary estimate for all drugs (ES = 0.27), whereas vilazodone’s MADRS effect size (ES = 0.41) was above the average for all drugs (ES = 0.30). This is a peculiar inconsistency, because if vilazodone was truly more efficacious than other drugs, this should show in an above-average score for both HDRS-17 and MADRS, but not in a very low HDRS-17 score and in a high MADRS score. This inconsistency might be explained by the low proportion of published vilazodone trials using the HDRS-17 (2 of 6; 33%), whereas all three trials using the MADRS were published (100%). It is known that the trials published in the scientific literature exaggerate efficacy estimates [28, 29]. The significant effect size difference between HDRS-17 and MADRS for vilazodone should thus be interpreted with caution, as this is likely a methodological artefact.

Previous research tried to minimise biases assumed to underlie the full HDRS scale by focusing on selected items. For instance, Bech developed a HDRS subscale that contains only core depression symptoms [30]. In a recent review he reported various trials where this subscale produced higher effect size estimates than the full HDRS scale [27], but these findings appear to be systematically biased due to selective reporting. Indeed, with an unweighted mean of 0.44 the summary effect size for the Bech-subscale reported by Bech [27] is considerably larger than in the comprehensive meta-analysis by Hieronymus et al. [15], where it was estimated with 0.35.

Hieronymus et al. [15] chose an even more reductionistic approach. They focused exclusively on a single item of the HDRS—the mood item—and reported higher effect sizes for the mood item compared to the total score (ES: 0.40 vs. 0.27). However, focusing on the mood item of the HDRS has major limitations. Contrary to the MADRS and the Bech-subscale, the psychometric properties of the mood item as a stand-alone outcome measure have never been validated. Moreover, simply because a specific outcome is more responsive to antidepressant treatment effects does not imply that it is a valid measure of depression [31]. It is not clear whether the mood item adequately captures the multifaceted construct of depression, as other relevant core depression symptoms such as suicidality, fatigue, feelings of guilt or loss of interest are ignored. This is particularly relevant, given that patients rate depressed mood as less important than for instance fatigue or loss of interest [32].

### Limitations

This study is not without limitations. First, it is important to note that the MADRS was applied in relatively few trials and comparisons between MADRS and HDRS-17 for individual drugs are thus limited to bupropion, duloxetine, escitalopram, paroxetine, venlafaxine, and vilazodone. However, it is unlikely that this restriction has introduced systematic bias. Gartlehner et al. [33] conclude that differences in efficacy estimates between new-generation antidepressants are not practically relevant, while Cipriani et al. [6] estimate the efficacy of these particular drugs as average (bupropion, duloxetine, vilazodone) or even above average (escitalopram, paroxetine, venlafaxine). Second, confidence intervals for some individual drugs are rather wide. Therefore, it cannot be ruled out that for some drugs the true effect might be considerably larger or smaller. However, given that differences in effect size estimates between individual drugs are very small [6, 33], we suggest that the summary estimate of 0.30 for the MADRS accurately reflects the true average treatment effect of all new-generation antidepressants. Third, it should be kept in mind that most trials included in this meta-analysis were sponsored by the drug manufacturers and that efficacy estimates based on industry-sponsored trials are likely overestimations due to various systematic method biases [9, 11, 34]. Fourth, most studies were rated at high or unclear risk of bias with respect to allocation concealment and blinding of participants [11], which also results in exaggerated effect size estimates in trials of subjective outcomes such as the HDRS or MADRS [35, 36]. We thus must remain mindful that the reported effect sizes represent upper-bound estimates.

## Conclusions

The effect size estimates reported in recent meta-analyses of antidepressants that were mostly based on the highly criticised HDRS-17 do not appear to underestimate the drugs treatment effects. When efficacy estimates are based on the MADRS, the preferred rating scale for depression that is particularly sensitive to change in core depression symptoms, the effect size estimates do not differ meaningfully. Moreover, we found that the average drug-placebo difference in raw scores for both the HDRS-17 and MADRS fall short of the cut-offs for a minimally important improvement. Prominent claims that the HDRS has significantly underestimated antidepressants’ true efficacy are thus not based on convincing evidence. A more plausible explanation would be that the efficacy of antidepressants is indeed poor and of questionable importance to the average patient.

## Supporting information

S1 Checklist PRISMA 2009 checklist. (DOC)

S1 File (PDF)
