# Supplementary material for: Efficacy of new-generation antidepressants assessed with the Montgomery-Asberg Depression Rating Scale, the gold standard clinician rating scale: A meta-analysis of randomised placebo-controlled trials
Source: PLoS One. 2020 Feb 26;15(2):e0229381. doi: 10.1371/journal.pone.0229381 (PMC7043778; doi:10.1371/journal.pone.0229381)
Supplement: S1 File — (PDF) [file pone.0229381.s002.pdf]

Supplement to paper
"Efficacy of new-generation antidepressants assessed with the Montgomery-Asberg Depression Rating Scale, the gold standard clinician rating scale: A meta-analysis of randomized controlled trials"

Michael P. Hengartner, Janus C. Jacobsen, Anders Sørensen, Martin Plöderl

October 25, 2019

Contents

1 Meta-analysis across all antidepressants 2

2 Meta-analyses for individual Antidepressants 4

3 Meta-analyses with raw mean differences 10

1 Meta-analysis across all antidepressants

Study or Subgroup	Experimental Mean	Experimental SD	Experimental Total	Control Mean	Control SD	Control Total	Weight (fixed)	Weight (random)	Std. Mean Difference IV, Fixed + Random, 95% CI
instrument = HAMD17									
CL3−20098−022	13.90	7.9000	262	15.90	8.6000	147	0.9%	0.9%	−0.24 [−0.45; −0.04]
CL3−20098−023	12.60	8.0500	278	13.80	8.0000	137	0.9%	0.8%	−0.15 [−0.35; 0.06]
Kennedy2006 (CL3−20098−043)	14.10	7.7000	106	16.50	7.4000	105	0.5%	0.7%	−0.32 [−0.59; −0.05]
Olie2007 (CL3−20098−042)	13.90	7.7000	116	17.00	7.9000	119	0.6%	0.7%	−0.40 [−0.65; −0.14]
Stahl2010 (CAGO178A2302)	15.45	8.1500	319	17.10	7.9200	163	1.1%	0.9%	−0.20 [−0.39; −0.01]
Zajecka2010 (CAGO178A2301) (NC	15.00	7.7400	317	16.60	8.4000	167	1.1%	0.9%	−0.20 [−0.39; −0.01]
CL3−20098−024	12.60	7.8000	441	13.40	8.4000	158	1.2%	0.9%	−0.10 [−0.28; 0.08]
CL3−20098−026	−10.60	8.1000	105	−9.60	7.3000	105	0.5%	0.7%	−0.13 [−0.40; 0.14]
CL3−20098−070	−13.40	8.3000	148	−10.70	7.8000	70	0.5%	0.7%	−0.33 [−0.62; −0.04]
CAGO178A2303 (NCT00463242)	15.55	7.4550	325	17.30	7.9200	158	1.1%	0.9%	−0.23 [−0.42; −0.04]
Loo2002 (CL2−014)	12.93	8.3000	279	15.34	8.8700	136	0.9%	0.8%	−0.28 [−0.49; −0.08]
Kennedy2014 (EudraCT 2009−0112	13.95	6.4500	274	18.70	7.3000	141	0.9%	0.8%	−0.70 [−0.91; −0.49]
Heun2013 (ISRCTN5750736O)	13.40	7.5000	148	16.10	7.6000	70	0.5%	0.7%	−0.36 [−0.64; −0.07]
Shipley1981	9.30	10.9000	53	31.60	14.9000	23	0.1%	0.3%	−1.81 [−2.38; −1.23]
Paykel1988	5.67	7.5200	45	9.46	7.5200	55	0.2%	0.5%	−0.50 [−0.90; −0.10]
Carman1991	−13.50	7.7600	48	−7.50	7.7600	48	0.2%	0.5%	−0.77 [−1.18; −0.35]
Katz1993a	−12.90	7.7600	95	−8.70	7.7600	94	0.5%	0.7%	−0.54 [−0.83; −0.25]
Katz1993b	−13.70	7.7600	93	−8.00	7.7600	104	0.5%	0.7%	−0.73 [−1.02; −0.44]
Wilcox1994	11.70	7.5200	39	17.40	7.5200	42	0.2%	0.4%	−0.75 [−1.20; −0.30]
Mynors−Wallis1995	10.30	6.5000	27	13.80	5.7000	26	0.1%	0.3%	−0.56 [−1.11; −0.01]
Reimherr1998 (WELL203, FDA203)	−10.18	8.0700	113	−8.06	8.1400	117	0.6%	0.7%	−0.26 [−0.52; 0.00]
Tomarken2004	12.60	7.5200	9	16.30	7.5200	9	0.0%	0.1%	−0.47 [−1.41; 0.47]
Clayton2006b (WELL AK130927)	−13.70	7.5050	267	−12.10	7.9800	130	0.9%	0.8%	−0.21 [−0.42; 0.00]
Clayton2006a (WELL AK130926)	−13.00	8.0100	262	−11.90	7.8600	126	0.9%	0.8%	−0.14 [−0.35; 0.07]
Study 205 (FDA) (WELL 205)	−9.30	7.8800	336	−8.30	7.8800	116	0.9%	0.8%	−0.13 [−0.34; 0.08]
AK1102365	15.40	8.3200	160	15.80	8.0400	155	0.8%	0.8%	−0.05 [−0.27; 0.17]
Montgomery1992 (Study 89303 FD	−12.41	8.7900	129	−10.56	7.7600	64	0.4%	0.6%	−0.22 [−0.52; 0.08]
Liebowitz2008 (Study 332, NCT0	−11.20	7.1300	297	−9.53	7.1000	150	1.0%	0.9%	−0.23 [−0.43; −0.04]
Boyer2008 (Study 333, NCT00300	−13.41	7.5500	322	−10.78	7.6100	161	1.1%	0.9%	−0.35 [−0.54; −0.16]
Lieberman2008a (Study 309)(EUC	−13.80	7.7800	127	−12.50	7.7800	120	0.6%	0.7%	−0.17 [−0.42; 0.08]
Lieberman2008b (Study 317)	−12.60	8.1500	115	−9.78	8.1600	125	0.6%	0.7%	−0.34 [−0.60; −0.09]
Tourian2009 (NCT00384033)	−10.22	7.7600	455	−8.70	7.7600	160	1.2%	0.9%	−0.20 [−0.38; −0.02]
Dunlop2011 (NCT00824291)	−12.61	7.6000	285	−10.50	7.1000	142	1.0%	0.9%	−0.28 [−0.49; −0.08]
Clayton2013 (NCT01121484)	−9.90	7.7600	216	−8.10	7.7600	216	1.1%	0.9%	−0.23 [−0.42; −0.04]
Iwata2013 (NCT00798707)	13.00	12.3200	236	14.60	12.2600	231	1.2%	0.9%	−0.13 [−0.31; 0.05]
Liebowitz2013 (NCT00863798)	14.11	6.5600	224	14.70	6.5500	223	1.1%	0.9%	−0.09 [−0.28; 0.10]
Clayton2015	−11.48	6.7000	592	−9.71	6.8600	294	2.0%	1.0%	−0.26 [−0.40; −0.12]
DeMartinis2007 (Study 306, NCT	−10.50	7.0400	114	−7.70	6.8400	118	0.6%	0.7%	−0.40 [−0.66; −0.14]
Goldstein2002 (HMAQ − Study Gr	−8.74	6.8650	101	−6.61	6.7400	66	0.4%	0.6%	−0.31 [−0.62; 0.00]
Study F1J−MC−HMAQ − Study Grou	−7.82	6.8750	118	−7.10	6.9600	72	0.5%	0.7%	−0.10 [−0.40; 0.19]
Goldstein2004a (HMAT − Study G	−6.83	6.4100	258	−4.78	6.4200	89	0.7%	0.8%	−0.32 [−0.56; −0.08]
Goldstein2004b (HMAT − Study	−7.12	7.4700	254	−4.99	7.6000	88	0.7%	0.8%	−0.28 [−0.53; −0.04]
Detke2002a (HMBH − Study Grou	−10.91	7.7000	121	−6.05	7.4000	115	0.6%	0.7%	−0.64 [−0.90; −0.38]
Detke2002b (HMBH − Study Group	−10.46	7.8700	123	−8.29	7.8100	136	0.6%	0.8%	−0.28 [−0.52; −0.03]
Detke2004 (HMAY Study Group A)	−10.73	5.5150	271	−8.07	5.5000	93	0.7%	0.8%	−0.48 [−0.72; −0.24]
Raskin2007 (HMBV) (NCT00062673	−6.49	6.8100	201	−3.72	6.3600	102	0.7%	0.8%	−0.41 [−0.66; −0.17]
Nierenberg2007 (F1J−MC−HMCR, N	−7.42	6.7800	547	−5.97	6.7900	137	1.1%	0.9%	−0.21 [−0.40; −0.03]
Perahia2006 (HMAY − Study Grou	−10.98	5.6650	292	−10.13	5.5700	99	0.7%	0.8%	−0.15 [−0.38; 0.08]
Higuchi2009	−9.82	6.4750	295	−8.30	5.8000	145	1.0%	0.9%	−0.24 [−0.44; −0.04]
Cutler2009	−12.37	9.1700	141	−10.26	9.1700	152	0.7%	0.8%	−0.23 [−0.46; 0.00]
Hirayasu2011a	9.30	7.1000	197	9.30	6.6000	100	0.7%	0.8%	0.00 [−0.24; 0.24]
Griebel2012 (Study DF15878) (N	−9.27	7.5300	81	−7.70	7.5700	71	0.4%	0.6%	−0.21 [−0.53; 0.11]
Mischoulon2014 (NCT00101452)	11.65	10.7500	65	12.31	8.6500	60	0.3%	0.6%	−0.07 [−0.42; 0.28]
Heiligenstein1994	−12.30	7.7600	45	−8.52	7.7600	43	0.2%	0.4%	−0.48 [−0.91; −0.06]
Tollefson1995	14.00	7.7000	326	15.70	7.4000	329	1.7%	1.0%	−0.22 [−0.38; −0.07]
McGrath2000	7.70	5.6000	49	10.30	6.3000	52	0.3%	0.5%	−0.43 [−0.83; −0.04]
Fava2005	13.30	7.3000	47	12.60	6.4000	43	0.2%	0.5%	0.10 [−0.31; 0.51]
Norton1984	11.57	7.5200	33	11.59	7.5200	25	0.1%	0.3%	−0.00 [−0.52; 0.52]
Montgomery2013 (F02695LP202, E	−14.90	7.4800	276	−11.50	7.6600	277	1.4%	0.9%	−0.45 [−0.62; −0.28]
Gommoll2014 (LVM−MD−02, NCT009	−9.90	9.2300	174	−9.20	8.0700	181	0.9%	0.8%	−0.08 [−0.29; 0.13]
Asnis2013 (LVM−MD−01, NCT00969	−10.30	9.2500	529	−8.40	8.8600	175	1.3%	0.9%	−0.21 [−0.38; −0.04]
Sambunaris2014 (LVM−MD−03, NCT	−9.60	7.3300	215	−7.50	7.3100	214	1.1%	0.9%	−0.29 [−0.48; −0.10]
MIR 84062 (FDA)	8.70	9.4500	30	8.40	9.6000	15	0.1%	0.3%	0.03 [−0.59; 0.65]
Kinoshita2009	−13.00	7.1000	200	−10.40	7.5000	70	0.5%	0.7%	−0.36 [−0.63; −0.09]
003−048	12.30	7.0800	179	13.20	7.7300	59	0.4%	0.7%	−0.12 [−0.42; 0.17]
D'Amico1990 (FDA 030−A2−0007)	−12.00	8.0500	87	−9.80	8.0500	47	0.3%	0.5%	−0.27 [−0.63; 0.08]
Feighner1998	−12.20	9.1400	58	−7.70	8.4500	59	0.3%	0.5%	−0.51 [−0.88; −0.14]
Fontaine1994 (FDA 03A0A−003)	−9.60	8.8900	90	−6.80	8.8900	45	0.3%	0.5%	−0.31 [−0.67; 0.05]
Mendels1995 (FDA 03A0A−004B)	−11.40	8.8500	156	−9.50	8.8500	75	0.5%	0.7%	−0.21 [−0.49; 0.06]
Rickels1994 (FDA CN104−005)	−12.00	7.6400	86	−8.20	7.6400	90	0.4%	0.6%	−0.50 [−0.80; −0.20]
Rickels1995 (FDA CN104−006−1)	−8.00	7.3000	41	−8.60	7.3000	36	0.2%	0.4%	0.08 [−0.37; 0.53]
Cohn1996 (FDA CN104−006−2)	−12.10	7.6000	39	−9.20	7.6000	42	0.2%	0.4%	−0.38 [−0.82; 0.06]
CN104−002 (FDA)	−10.80	7.8600	57	−8.20	7.8600	57	0.3%	0.5%	−0.33 [−0.70; 0.04]
03A0A−004A (FDA)	−8.90	7.9600	153	−8.90	7.9600	77	0.5%	0.7%	0.00 [−0.27; 0.27]
Edwards1989 (MD/PAR/009 PAR−27	−8.80	10.5000	21	−4.50	8.8000	20	0.1%	0.3%	−0.43 [−1.05; 0.19]
Golden2002a (29060/448)	−11.90	8.1700	206	−9.90	8.0400	101	0.7%	0.8%	−0.25 [−0.48; −0.01]
Golden2002b (29060/449)	−12.70	8.2000	218	−10.20	8.1800	110	0.7%	0.8%	−0.30 [−0.53; −0.07]
Rapaport2003 (PAR487)	10.00	7.2600	210	12.60	7.3400	109	0.7%	0.8%	−0.36 [−0.59; −0.12]
Trivedi2004 (29060/810)	−12.40	7.1700	143	−10.00	7.1500	142	0.7%	0.8%	−0.33 [−0.57; −0.10]
NKD20006 (NCT00048204)	−11.10	7.9000	117	−10.90	7.8000	118	0.6%	0.7%	−0.03 [−0.28; 0.23]
Rapaport2009 (BRL−29060/874) (	−12.11	8.0000	173	−8.85	8.0500	178	0.9%	0.8%	−0.41 [−0.62; −0.19]
Higuchi2011 (PCR112810, NCT008	−12.65	7.3900	241	−10.40	8.1100	171	1.0%	0.9%	−0.29 [−0.49; −0.09]
Keller2006c (Study062) (NCT000	−17.25	8.0500	161	−14.00	8.8700	154	0.8%	0.8%	−0.38 [−0.61; −0.16]
Griebel2012b (Study DF15879) (	−16.10	7.4000	79	−12.75	6.9700	76	0.4%	0.6%	−0.46 [−0.78; −0.14]
Studie009 (CTN009−FCE20124)	14.38	8.9400	24	15.09	8.5200	23	0.1%	0.3%	−0.08 [−0.65; 0.49]
Reimherr1990 (SER 104 − FDA)	−12.15	8.1050	286	−8.16	7.8500	141	0.9%	0.8%	−0.50 [−0.70; −0.29]
Lydiard1997	−11.95	6.8500	223	−8.80	6.9700	115	0.8%	0.8%	−0.46 [−0.68; −0.23]
Schneider2003	14.00	6.5000	360	14.80	6.3000	368	1.8%	1.0%	−0.12 [−0.27; 0.02]
Moscovitch2004	−9.36	7.7100	93	−6.87	8.0000	93	0.5%	0.7%	−0.32 [−0.60; −0.03]
Barber2011	13.70	9.0000	55	14.00	10.7000	50	0.3%	0.5%	−0.03 [−0.41; 0.35]
Binnemann2008 (NCT00143091)	−13.42	7.6100	30	−10.18	7.5700	31	0.2%	0.4%	−0.42 [−0.93; 0.09]
Fabre1995 (SER 103 FDA)	−9.87	8.5700	261	−7.60	7.5000	86	0.7%	0.8%	−0.27 [−0.52; −0.03]
SER 315 (FDA)	−10.25	8.0050	146	−7.80	8.0000	73	0.5%	0.7%	−0.31 [−0.59; −0.02]
Mao2015 (NCT01098318)	−8.20	10.1000	19	−4.60	8.6600	18	0.1%	0.2%	−0.37 [−1.02; 0.28]
Davidson2002 (HDTSG) (NCT00005	−10.53	7.5200	109	−9.20	7.2200	116	0.6%	0.7%	−0.18 [−0.44; 0.08]
Olie1997	−11.70	8.1000	129	−7.70	8.1000	129	0.6%	0.8%	−0.49 [−0.74; −0.24]
PZ/109	13.30	7.8000	104	13.60	8.0000	103	0.5%	0.7%	−0.04 [−0.31; 0.23]
PZ/111	13.10	6.9500	214	13.80	7.5000	105	0.7%	0.8%	−0.10 [−0.33; 0.14]
Sheehan2009b (NCT00775203)	11.80	8.0000	202	13.20	8.1000	204	1.0%	0.9%	−0.17 [−0.37; 0.02]
Zhang2014	10.80	6.4000	183	13.80	6.9000	180	0.9%	0.8%	−0.45 [−0.66; −0.24]
Learned2012a Study1 (EUCTR2005	−14.00	6.3600	133	−12.70	7.6800	126	0.7%	0.8%	−0.18 [−0.43; 0.06]
Higuchi 2014(NCT01441440)	−10.57	6.5600	351	−9.25	6.5100	184	1.2%	0.9%	−0.20 [−0.38; −0.02]
Rickels2009 (GNSC−04−DP−02 FDA	−10.40	8.4400	198	−8.60	8.4600	199	1.0%	0.9%	−0.21 [−0.41; −0.02]
Khan2011 (CLDA−07−DP−02 FDA) (	−10.70	10.6400	231	−9.10	10.6600	232	1.2%	0.9%	−0.15 [−0.33; 0.03]
FDA 245 (EMD 68 843−010)	−10.40	7.4050	196	−10.20	7.9600	99	0.7%	0.8%	−0.03 [−0.27; 0.22]
FDA 246 (SB 659746−003)	12.55	7.5200	356	13.10	7.5200	128	1.0%	0.9%	−0.07 [−0.28; 0.13]
FDA 247 (SB 659746−014)	12.80	5.9900	107	13.70	5.9900	110	0.5%	0.7%	−0.15 [−0.42; 0.12]
FDA 248 (SB 659746−002)	12.15	7.5200	265	12.20	7.5200	128	0.9%	0.8%	−0.01 [−0.22; 0.20]
NCT01355081(japicCTI−111492, U	−9.05	6.3900	240	−8.40	6.3800	122	0.8%	0.8%	−0.10 [−0.32; 0.12]
Total (fixed effect, 95% CI)			19796			12603	74.1%	−−	−0.25 [−0.28; −0.23]
Total (random effects, 95% CI)							−−	77.4%	−0.27 [−0.30; −0.23]
Heterogeneity: $Tau^2 = 0.0292$; $Chi^2 = 195.95$, df = 108 ($P < 0.01$); $I^2 = 45\%$									
instrument = MADRS									
Hewett2009 (WXL101497)	−16.55	9.1400	372	−13.50	9.1200	197	1.3%	0.9%	−0.33 [−0.51; −0.16]
Hewett2010a (AK130940) (NCT000	−13.90	9.5600	210	−12.40	9.7100	204	1.0%	0.9%	−0.16 [−0.35; 0.04]
Hewett2010b (AK130939)	−17.00	10.5600	193	−13.20	10.6400	186	0.9%	0.9%	−0.36 [−0.56; −0.15]
Koshino2013 (NCT01138007)	−12.90	10.4200	188	−13.90	10.5000	186	0.9%	0.9%	0.10 [−0.11; 0.30]
Study 89306 (FDA)	−16.99	13.2200	185	−15.95	13.2200	88	0.6%	0.7%	−0.08 [−0.33; 0.18]
NCT01145755	−14.03	13.2200	45	−14.16	13.6600	40	0.2%	0.4%	0.01 [−0.42; 0.44]
Lepola2003 (ESC 99003)	−14.30	8.7700	314	−12.10	8.6900	154	1.0%	0.9%	−0.25 [−0.44; −0.06]
Ninan2003 (poster SCT−MD−26)	−13.30	10.7600	143	−10.00	11.0600	151	0.7%	0.8%	−0.30 [−0.53; −0.07]
Wade2002 (ESC Study 99001 − FD	14.30	9.1000	188	16.70	9.1000	189	0.9%	0.9%	−0.26 [−0.47; −0.06]
Kasper2005a (Study 99024)	−15.15	9.9600	337	−15.00	9.9600	180	1.2%	0.9%	−0.02 [−0.20; 0.17]
Hirayasu2011b	15.75	10.2750	360	18.30	10.1000	124	0.9%	0.8%	−0.25 [−0.45; −0.04]
Kasper2012 (NCT00807248)	−19.00	10.6500	140	−13.40	9.2700	71	0.5%	0.7%	−0.55 [−0.84; −0.26]
Bakish2014 (LVM−MD−10, NCT0137	−14.50	10.7700	372	−11.30	10.4700	185	1.2%	0.9%	−0.30 [−0.48; −0.12]
Jefferson2000 (29060/785)	−14.47	10.6450	296	−12.10	11.0500	101	0.8%	0.8%	−0.22 [−0.45; 0.01]
Learned2012b Study2 (NCT004206	−19.11	10.3000	166	−14.95	10.2300	156	0.8%	0.8%	−0.40 [−0.63; −0.18]
Doogan1994	−15.40	9.9600	83	−12.10	9.9600	90	0.4%	0.6%	−0.33 [−0.63; −0.03]
Brunoni2012	21.67	13.1400	30	24.73	8.6500	30	0.2%	0.4%	−0.27 [−0.78; 0.24]
Lecrubier1997	−16.93	8.2200	72	−14.07	8.2200	76	0.4%	0.6%	−0.35 [−0.67; −0.02]
Croft2014 (NCT01473394)	14.60	11.1300	253	20.10	11.1100	252	1.2%	0.9%	−0.49 [−0.67; −0.32]
Mathews2015 (NCT01473381)	−16.70	9.9600	852	−14.31	9.9600	281	2.1%	1.0%	−0.24 [−0.38; −0.11]
Pomara2013	−16.10	9.9600	255	−11.00	9.9600	253	1.2%	0.9%	−0.51 [−0.69; −0.33]
Boulenger2014 (13267A, NCT0114	−19.58	9.4550	446	−11.70	9.5500	158	1.1%	0.9%	−0.83 [−1.02; −0.64]
Jacobsen2015 (316, NCT01163266	−13.69	9.3000	246	−10.77	9.5100	139	0.9%	0.8%	−0.31 [−0.52; −0.10]
Mahableshwarkar2015a (315, NCT	−15.92	9.4350	340	−12.83	9.4700	129	0.9%	0.8%	−0.33 [−0.53; −0.12]
Mahableshwarkar2015b (317, NCT	−13.51	11.6800	236	−12.87	11.7100	126	0.8%	0.8%	−0.05 [−0.27; 0.16]
NCT01255787 (EUCTR2010−022257−	−15.37	9.5900	438	−13.99	9.5900	150	1.1%	0.9%	−0.14 [−0.33; 0.04]
McIntyre2014 (NCT01422213)	−16.56	8.3300	355	−10.85	8.2200	165	1.1%	0.9%	−0.69 [−0.88; −0.50]
Mahableshwarkar2015c (NCT01564	−15.30	9.4150	362	−12.50	9.0500	167	1.1%	0.9%	−0.30 [−0.48; −0.12]
Total (fixed effect, 95% CI)			7477			4228	25.9%	−−	−0.30 [−0.34; −0.27]
Total (random effects, 95% CI)							−−	22.6%	−0.30 [−0.38; −0.22]
Heterogeneity: $Tau^2 = 0.0309$; $Chi^2 = 101.89$, df = 27 ($P < 0.01$); $I^2 = 74\%$									
**Total (fixed effect, 95% CI)**			**27273**			**16831**	**100.0%**	**−−**	**−0.27 [−0.29; −0.25]**
**Total (random effects, 95% CI)**							**−−**	**100.0%**	**−0.27 [−0.31; −0.24]**
Heterogeneity: $Tau^2 = 0.0293$; $Chi^2 = 302.51$, df = 136 ($P < 0.01$); $I^2 = 55\%$									
Residual heterogeneity: $Tau^2 = NA$; $Chi^2 = 297.83$, df = 135 ($P < 0.01$); $I^2 = 55\%$									

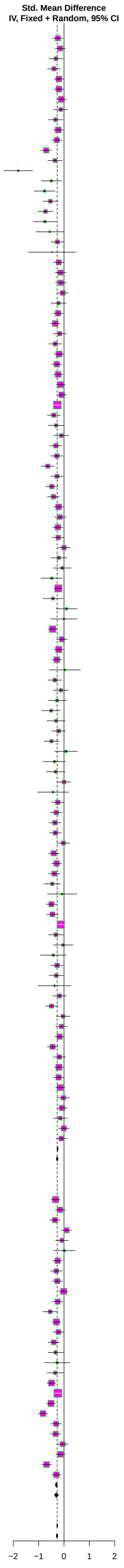

Figure 1: Meta-analytic results for all AD, separated by type of instrument.

2 Meta-analyses for individual Antidepressants

Study or Subgroup	Experimental Mean	SD	Total	Control Mean	SD	Total	Weight (fixed)	Weight (random)	Std. Mean Difference IV, Fixed + Random, 95% CI
instrument = HAMD17									
Reimherr1998 (WELL203, FDA203)	−10.18	8.0700	113	−8.06	8.1400	117	8.8%	10.1%	−0.26 [−0.52; 0.00]
Tomarken2004	12.60	7.5200	9	16.30	7.5200	9	0.7%	1.1%	−0.47 [−1.41; 0.47]
Clayton2006b (WELL AK130927)	−13.20	6.9400	134	−12.10	7.9800	130	10.2%	11.1%	−0.15 [−0.39; 0.09]
Clayton2006a (WELL AK130926)	−13.10	7.9500	129	−11.90	7.8600	126	9.8%	10.8%	−0.15 [−0.40; 0.09]
Study 205 (FDA) (WELL 205)	−9.30	7.8800	336	−8.30	7.8800	116	13.3%	13.0%	−0.13 [−0.34; 0.08]
AK1102365	15.40	8.3200	160	15.80	8.0400	155	12.2%	12.3%	−0.05 [−0.27; 0.17]
Total (fixed effect, 95% CI)			881			653	54.9%	−−	−0.14 [−0.25; −0.04]
Total (random effects, 95% CI)							−−	58.4%	−0.15 [−0.23; −0.06]
Heterogeneity: $Tau^2 = 0.0040$; $Chi^2 = 1.98$, df = 5 (P = 0.85); $I^2 = 0\%$									
instrument = MADRS									
Hewett2009 (WXL101497)	−16.00	9.0300	187	−13.50	9.1200	197	14.7%	13.7%	−0.27 [−0.48; −0.07]
Hewett2010a (AK130940) (NCT000	−13.90	9.5600	210	−12.40	9.7100	204	15.9%	14.3%	−0.16 [−0.35; 0.04]
Koshino2013 (NCT01138007)	−12.90	10.4200	188	−13.90	10.5000	186	14.4%	13.6%	0.10 [−0.11; 0.30]
Total (fixed effect, 95% CI)			585			587	45.1%	−−	−0.11 [−0.23; 0.00]
Total (random effects, 95% CI)							−−	41.6%	−0.11 [−0.58; 0.36]
Heterogeneity: $Tau^2 = 0.0247$; $Chi^2 = 6.73$, df = 2 (P = 0.03); $I^2 = 70\%$									
**Total (fixed effect, 95% CI)**			**1466**			**1240**	**100.0%**	**−−**	**−0.13 [−0.21; −0.05]**
**Total (random effects, 95% CI)**							**−−**	**100.0%**	**−0.13 [−0.23; −0.04]**
Heterogeneity: $Tau^2 = 0.0091$; $Chi^2 = 8.85$, df = 8 (P = 0.36); $I^2 = 10\%$									
Residual heterogeneity: $Tau^2 = NA$; $Chi^2 = 8.71$, df = 7 (P = 0.27); $I^2 = 20\%$									

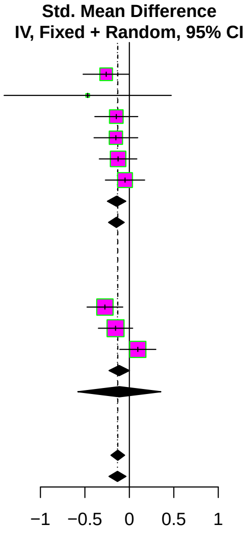

Figure 2: Meta-analytic results for bupropion, separated by type of instrument.

Study or Subgroup	Experimental Mean	SD	Total	Control Mean	SD	Total	Weight (fixed)	Weight (random)	Std. Mean Difference IV, Fixed + Random, 95% CI
instrument = HAMD17									
Tourian2009 (NCT00384033)	−10.30	7.7600	157	−8.70	7.7600	160	7.5%	6.5%	−0.21 [−0.43; 0.02]
Goldstein2002 (HMAQ − Study Gr	−9.73	6.8400	68	−6.61	6.7400	66	3.1%	4.7%	−0.46 [−0.80; −0.11]
Study F1J−MC−HMAQ − Study Grou	−8.00	6.7500	81	−7.10	6.9600	72	3.6%	5.0%	−0.13 [−0.45; 0.19]
Goldstein2004a (HMAT − Study G	−6.25	6.3800	171	−4.78	6.4200	89	5.6%	5.9%	−0.23 [−0.49; 0.03]
Goldstein2004b (HMAT − Study	−8.02	7.4200	170	−4.99	7.6000	88	5.4%	5.9%	−0.40 [−0.66; −0.14]
Detke2002a (HMBH − Study Grou	−10.91	7.7000	121	−6.05	7.4000	115	5.4%	5.8%	−0.64 [−0.90; −0.38]
Detke2002b (HMBH − Study Group	−10.46	7.8700	123	−8.29	7.8100	136	6.1%	6.1%	−0.28 [−0.52; −0.03]
Detke2004 (HMAY Study Group A)	−10.64	5.5000	186	−8.07	5.5000	93	5.8%	6.0%	−0.47 [−0.72; −0.21]
Raskin2007 (HMBV) (NCT00062673	−6.49	6.8100	201	−3.72	6.3600	102	6.3%	6.2%	−0.41 [−0.66; −0.17]
Nierenberg2007 (F1J−MC−HMCR, N	−7.61	6.9400	273	−5.97	6.7900	137	8.7%	6.7%	−0.24 [−0.44; −0.03]
Perahia2006 (HMAY − Study Grou	−11.35	5.6200	195	−10.13	5.5700	99	6.2%	6.1%	−0.22 [−0.46; 0.03]
Higuchi2009	−10.25	6.0500	147	−8.30	5.8000	145	6.9%	6.3%	−0.33 [−0.56; −0.10]
Cutler2009	−12.37	9.1700	141	−10.26	9.1700	152	6.9%	6.3%	−0.23 [−0.46; 0.00]
**Total (fixed effect, 95% CI)**			**2034**			**1454**	**77.6%**	**−−**	**−0.32 [−0.39; −0.25]**
**Total (random effects, 95% CI)**							**−−**	**77.5%**	**−0.32 [−0.40; −0.24]**
Heterogeneity: $Tau^2 = 0.0102$; $Chi^2 = 13.57$, df = 12 (P = 0.33); $I^2 = 12\%$									
instrument = MADRS									
NCT01145755	−14.03	13.2200	45	−14.16	13.6600	40	2.0%	3.8%	0.01 [−0.42; 0.44]
Boulenger2014 (13267A, NCT0114	−21.15	9.3000	146	−11.70	9.5500	158	6.4%	6.2%	−1.00 [−1.24; −0.76]
Mahableshwarkar2015a (315, NCT	−16.90	9.4800	115	−12.83	9.4700	129	5.7%	5.9%	−0.43 [−0.68; −0.17]
Mahableshwarkar2015c (NCT01564	−15.80	9.5700	187	−12.50	9.0500	167	8.3%	6.6%	−0.35 [−0.56; −0.14]
**Total (fixed effect, 95% CI)**			**493**			**494**	**22.4%**	**−−**	**−0.52 [−0.65; −0.40]**
**Total (random effects, 95% CI)**							**−−**	**22.5%**	**−0.46 [−1.12; 0.19]**
Heterogeneity: $Tau^2 = 0.1449$; $Chi^2 = 24.35$, df = 3 (P < 0.01); $I^2 = 88\%$									
**Total (fixed effect, 95% CI)**			**2527**			**1948**	**100.0%**	**−−**	**−0.36 [−0.42; −0.30]**
**Total (random effects, 95% CI)**							**−−**	**100.0%**	**−0.36 [−0.47; −0.25]**

Heterogeneity: $Tau^2 = 0.0357$; $Chi^2 = 45.76$, df = 16 (P < 0.01); $I^2 = 65\%$

Residual heterogeneity: Tau^2 = NA; $Chi^2 = 37.92$, df = 15 (P < 0.01); $I^2 = 60\%$

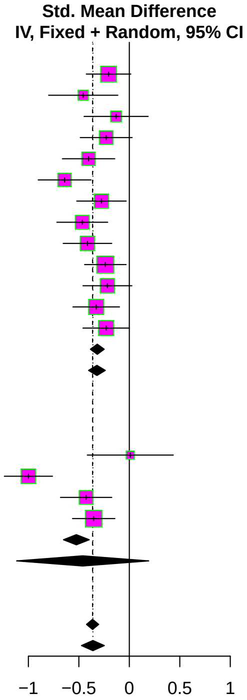

Figure 3: Meta-analytic results for duloxetine and separated by type of instrument.

Study or Subgroup	Experimental Mean	Experimental SD	Experimental Total	Control Mean	Control SD	Control Total	Weight (fixed)	Weight (random)	Std. Mean Difference IV, Fixed + Random, 95% CI
instrument = HAMD17									
Clayton2006b (WELL AK130927)	−14.20	8.0700	133	−12.10	7.9800	130	8.0%	8.3%	−0.26 [−0.50; −0.02]
Clayton2006a (WELL AK130926)	−12.90	8.0700	133	−11.90	7.8600	126	7.9%	8.3%	−0.13 [−0.37; 0.12]
Nierenberg2007 (F1J−MC−HMCR, N	−7.22	6.6200	274	−5.97	6.7900	137	11.2%	10.0%	−0.19 [−0.39; 0.02]
Hirayasu2011a	9.30	7.1000	197	9.30	6.6000	100	8.2%	8.4%	0.00 [−0.24; 0.24]
Griebel2012 (Study DF15878) (N	−9.27	7.5300	81	−7.70	7.5700	71	4.6%	5.9%	−0.21 [−0.53; 0.11]
Mischoulon2014 (NCT00101452)	11.65	10.7500	65	12.31	8.6500	60	3.8%	5.1%	−0.07 [−0.42; 0.28]
Total (fixed effect, 95% CI)			883			624	43.8%	−−	−0.15 [−0.25; −0.04]
Total (random effects, 95% CI)							−−	46.0%	−0.15 [−0.25; −0.04]
Heterogeneity: $Tau^2 = 0.0029$; $Chi^2 = 2.79$, df = 5 (P = 0.73); $I^2 = 0\%$									
instrument = MADRS									
Lepola2003 (ESC 99003)	−15.00	8.7100	155	−12.10	8.6900	154	9.4%	9.1%	−0.33 [−0.56; −0.11]
Ninan2003 (poster SCT−MD−26)	−13.30	10.7600	143	−10.00	11.0600	151	8.9%	8.9%	−0.30 [−0.53; −0.07]
Wade2002 (ESC Study 99001 − FD	14.30	9.1000	188	16.70	9.1000	189	11.5%	10.1%	−0.26 [−0.47; −0.06]
Kasper2005a (Study 99024)	−15.50	9.9600	173	−15.00	9.9600	180	10.8%	9.8%	−0.05 [−0.26; 0.16]
Hirayasu2011b	15.90	10.5500	239	18.30	10.1000	124	10.0%	9.4%	−0.23 [−0.45; −0.01]
Kasper2012 (NCT00807248)	−19.00	10.6500	140	−13.40	9.2700	71	5.6%	6.7%	−0.55 [−0.84; −0.26]
Total (fixed effect, 95% CI)			1038			869	56.2%	−−	−0.26 [−0.35; −0.17]
Total (random effects, 95% CI)							−−	54.0%	−0.27 [−0.43; −0.11]
Heterogeneity: $Tau^2 = 0.0147$; $Chi^2 = 8.22$, df = 5 (P = 0.14); $I^2 = 39\%$									
**Total (fixed effect, 95% CI)**			**1921**			**1493**	**100.0%**	**−−**	**−0.21 [−0.28; −0.14]**
**Total (random effects, 95% CI)**							**−−**	**100.0%**	**−0.21 [−0.30; −0.12]**
Heterogeneity: $Tau^2 = 0.0114$; $Chi^2 = 13.71$, df = 11 (P = 0.25); $I^2 = 20\%$									
Residual heterogeneity: Tau^2 = NA; $Chi^2 = 11.01$, df = 10 (P = 0.36); $I^2 = 9\%$									

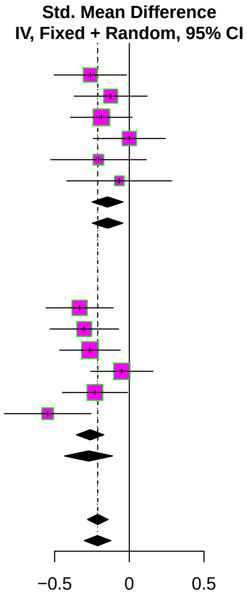

Figure 4: Meta-analytic results for escitalopram, separated by type of instrument.

Study or Subgroup	Experimental Mean	SD	Total	Control Mean	SD	Total	Weight (fixed)	Weight (random)	Std. Mean Difference IV, Fixed + Random, 95% CI
instrument = HAMD17									
CL3−20098−023	12.20	8.1000	137	13.80	8.0000	137	5.2%	5.2%	−0.20 [−0.44; 0.04]
CAGO178A2303 (NCT00463242)	14.00	7.5300	163	17.30	7.9200	158	6.0%	5.7%	−0.43 [−0.65; −0.20]
Loo2002 (CL2−014)	13.09	8.3700	144	15.34	8.8700	136	5.3%	5.2%	−0.26 [−0.50; −0.03]
Goldstein2004a (HMAT − Study G	−7.40	6.4400	87	−4.78	6.4200	89	3.3%	3.8%	−0.41 [−0.70; −0.11]
Goldstein2004b (HMAT − Study	−6.22	7.5200	84	−4.99	7.6000	88	3.3%	3.7%	−0.16 [−0.46; 0.14]
Detke2004 (HMAY Study Group A)	−10.83	5.5300	85	−8.07	5.5000	93	3.3%	3.7%	−0.50 [−0.80; −0.20]
Perahia2006 (HMAY − Study Grou	−10.61	5.7100	97	−10.13	5.5700	99	3.8%	4.1%	−0.08 [−0.36; 0.20]
Higuchi2009	−9.40	6.9000	148	−8.30	5.8000	145	5.6%	5.4%	−0.17 [−0.40; 0.06]
Edwards1989 (MD/PAR/009 PAR−27	−8.80	10.5000	21	−4.50	8.8000	20	0.8%	1.1%	−0.43 [−1.05; 0.19]
Golden2002a (29060/448)	−11.90	8.1700	206	−9.90	8.0400	101	5.2%	5.1%	−0.25 [−0.48; −0.01]
Golden2002b (29060/449)	−12.70	8.2000	218	−10.20	8.1800	110	5.5%	5.4%	−0.30 [−0.53; −0.07]
Rapaport2003 (PAR487)	10.00	7.2600	210	12.60	7.3400	109	5.4%	5.3%	−0.36 [−0.59; −0.12]
Trivedi2004 (29060/810)	−12.40	7.1700	143	−10.00	7.1500	142	5.4%	5.3%	−0.33 [−0.57; −0.10]
NKD20006 (NCT00048204)	−11.10	7.9000	117	−10.90	7.8000	118	4.5%	4.7%	−0.03 [−0.28; 0.23]
Rapaport2009 (BRL−29060/874) (	−12.11	8.0000	173	−8.85	8.0500	178	6.6%	6.0%	−0.41 [−0.62; −0.19]
Higuchi2011 (PCR112810, NCT008	−12.65	7.3900	241	−10.40	8.1100	171	7.6%	6.5%	−0.29 [−0.49; −0.09]
Keller2006c (Study062) (NCT000	−17.25	8.0500	161	−14.00	8.8700	154	5.9%	5.6%	−0.38 [−0.61; −0.16]
Griebel2012b (Study DF15879) (	−16.10	7.4000	79	−12.75	6.9700	76	2.9%	3.4%	−0.46 [−0.78; −0.14]
**Total (fixed effect, 95% CI)**			**2514**			**2124**	**85.5%**	**−−**	**−0.30 [−0.36; −0.24]**
**Total (random effects, 95% CI)**							**−−**	**85.4%**	**−0.30 [−0.36; −0.23]**
Heterogeneity: $Tau^2 = 0.0082$; $Chi^2 = 16.13$, df = 17 (P = 0.51); $I^2 = 0\%$									
instrument = MADRS									
Hirayasu2011b	15.60	10.0000	121	18.30	10.1000	124	4.7%	4.8%	−0.27 [−0.52; −0.02]
Jefferson2000 (29060/785)	−13.80	10.8300	97	−12.10	11.0500	101	3.8%	4.1%	−0.15 [−0.43; 0.12]
Learned2012b Study2 (NCT004206	−19.11	10.3000	166	−14.95	10.2300	156	6.0%	5.7%	−0.40 [−0.63; −0.18]
**Total (fixed effect, 95% CI)**			**384**			**381**	**14.5%**	**−−**	**−0.30 [−0.44; −0.15]**
**Total (random effects, 95% CI)**							**−−**	**14.6%**	**−0.29 [−0.60; 0.02]**
Heterogeneity: $Tau^2 = 0.0061$; $Chi^2 = 1.96$, df = 2 (P = 0.38); $I^2 = 0\%$									
**Total (fixed effect, 95% CI)**			**2898**			**2505**	**100.0%**	**−−**	**−0.30 [−0.35; −0.24]**
**Total (random effects, 95% CI)**							**−−**	**100.0%**	**−0.30 [−0.35; −0.24]**
Heterogeneity: $Tau^2 = 0.0076$; $Chi^2 = 18.08$, df = 20 (P = 0.58); $I^2 = 0\%$									
Residual heterogeneity: $Tau^2 = NA$; $Chi^2 = 18.08$, df = 19 (P = 0.52); $I^2 = 0\%$									

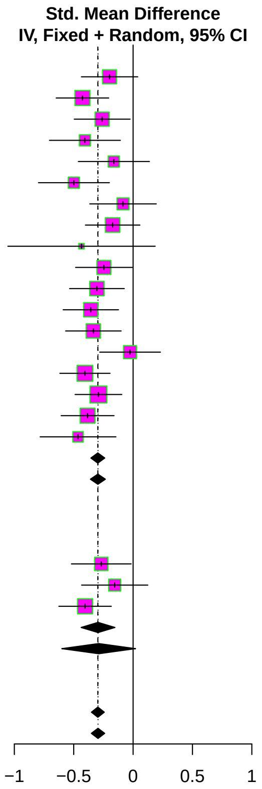

Figure 5: Meta-Analytic results for paroxetine, separated by type of instrument.

Study or Subgroup	Experimental Mean	Experimental SD	Experimental Total	Control Mean	Control SD	Control Total	Weight (fixed)	Weight (random)	Std. Mean Difference IV, Fixed + Random, 95% CI
instrument = HAMD17									
Lieberman2008a (Study 309)(EUC	−13.80	7.7800	127	−12.50	7.7800	120	11.6%	12.3%	−0.17 [−0.42; 0.08]
Lieberman2008b (Study 317)	−12.60	8.1500	115	−9.78	8.1600	125	11.2%	11.9%	−0.34 [−0.60; −0.09]
Learned2012a Study1 (EUCTR2005	−14.00	6.3600	133	−12.70	7.6800	126	12.2%	12.8%	−0.18 [−0.43; 0.06]
Higuchi 2014(NCT01441440)	−10.57	6.5600	351	−9.25	6.5100	184	22.7%	20.7%	−0.20 [−0.38; −0.02]
**Total (fixed effect, 95% CI)**			**726**			**555**	**57.7%**	**−−**	**−0.22 [−0.33; −0.11]**
**Total (random effects, 95% CI)**							**−−**	**57.8%**	**−0.22 [−0.34; −0.10]**
Heterogeneity: $Tau^2 = 0.0015$; $Chi^2 = 1.22$, df = 3 (P = 0.75); $I^2 = 0\%$									
instrument = MADRS									
Hewett2009 (WXL101497)	−17.10	9.2500	185	−13.50	9.1200	197	17.7%	17.2%	−0.39 [−0.59; −0.19]
Hewett2010b (AK130939)	−17.00	10.5600	193	−13.20	10.6400	186	17.6%	17.2%	−0.36 [−0.56; −0.15]
Lecrubier1997	−16.93	8.2200	72	−14.07	8.2200	76	6.9%	7.9%	−0.35 [−0.67; −0.02]
**Total (fixed effect, 95% CI)**			**450**			**459**	**42.3%**	**−−**	**−0.37 [−0.50; −0.24]**
**Total (random effects, 95% CI)**							**−−**	**42.2%**	**−0.37 [−0.43; −0.31]**
Heterogeneity: $Tau^2 = < 0.0001$; $Chi^2 = 0.08$, df = 2 (P = 0.96); $I^2 = 0\%$									
**Total (fixed effect, 95% CI)**			**1176**			**1014**	**100.0%**	**−−**	**−0.28 [−0.37; −0.20]**
**Total (random effects, 95% CI)**							**−−**	**100.0%**	**−0.28 [−0.37; −0.19]**
Heterogeneity: $Tau^2 = 0.0034$; $Chi^2 = 4.25$, df = 6 (P = 0.64); $I^2 = 0\%$									
Residual heterogeneity: $Tau^2 = NA$; $Chi^2 = 1.29$, df = 5 (P = 0.94); $I^2 = 0\%$									

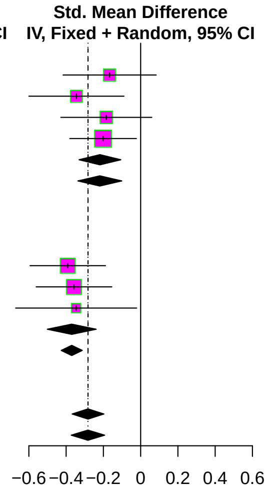

Figure 6: Meta-analytic results for venlafaxine, separated by type of instrument.

Study or Subgroup	Experimental Mean	Experimental SD	Experimental Total	Control Mean	Control SD	Control Total	Weight (fixed)	Weight (random)	Std. Mean Difference IV, Fixed + Random, 95% CI
instrument = HAMD17									
Rickels2009 (GNSC−04−DP−02 FDA	−10.40	8.4400	198	−8.60	8.4600	199	10.7%	11.3%	−0.21 [−0.41; −0.02]
Khan2011 (CLDA−07−DP−02 FDA) (	−10.70	10.6400	231	−9.10	10.6600	232	12.5%	11.7%	−0.15 [−0.33; 0.03]
FDA 245 (EMD 68 843−010)	−9.70	7.1400	104	−10.20	7.9600	99	5.5%	9.2%	0.07 [−0.21; 0.34]
FDA 246 (SB 659746−003)	12.80	7.5200	241	13.10	7.5200	128	9.1%	10.8%	−0.04 [−0.25; 0.17]
FDA 247 (SB 659746−014)	12.80	5.9900	107	13.70	5.9900	110	5.9%	9.4%	−0.15 [−0.42; 0.12]
FDA 248 (SB 659746−002)	12.15	7.5200	265	12.20	7.5200	128	9.4%	10.9%	−0.01 [−0.22; 0.20]
**Total (fixed effect, 95% CI)**			**1146**			**896**	**53.1%**	**−−**	**−0.10 [−0.18; −0.01]**
**Total (random effects, 95% CI)**							**−−**	**63.4%**	**−0.09 [−0.20; 0.01]**
Heterogeneity: $Tau^2 = 0.0044$; $Chi^2 = 4.12$, df = 5 (P = 0.53); $I^2 = 0\%$									
instrument = MADRS									
Croft2014 (NCT01473394)	14.60	11.1300	253	20.10	11.1100	252	13.3%	11.9%	−0.49 [−0.67; −0.32]
Mathews2015 (NCT01473381)	−16.91	9.9600	572	−14.31	9.9600	281	20.3%	12.8%	−0.26 [−0.40; −0.12]
Pomara2013	−16.10	9.9600	255	−11.00	9.9600	253	13.3%	11.9%	−0.51 [−0.69; −0.33]
**Total (fixed effect, 95% CI)**			**1080**			**786**	**46.9%**	**−−**	**−0.40 [−0.49; −0.30]**
**Total (random effects, 95% CI)**							**−−**	**36.6%**	**−0.41 [−0.77; −0.06]**
Heterogeneity: $Tau^2 = 0.0132$; $Chi^2 = 6.22$, df = 2 (P = 0.04); $I^2 = 68\%$									
**Total (fixed effect, 95% CI)**			**2226**			**1682**	**100.0%**	**−−**	**−0.24 [−0.30; −0.17]**
**Total (random effects, 95% CI)**							**−−**	**100.0%**	**−0.21 [−0.36; −0.05]**
Heterogeneity: $Tau^2 = 0.0312$; $Chi^2 = 31.26$, df = 8 (P < 0.01); $I^2 = 74\%$									
Residual heterogeneity: Tau^2 = NA; $Chi^2 = 10.34$, df = 7 (P = 0.17); $I^2 = 32\%$									

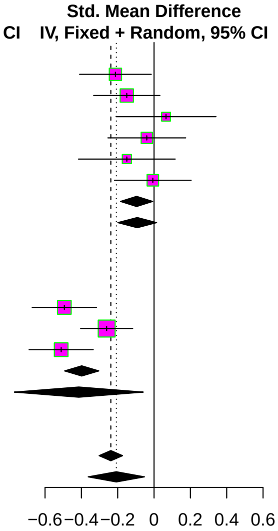

Figure 7: Meta-analytic results for vilazodone, separated by type of instrument.

3 Meta-analyses with raw mean differences

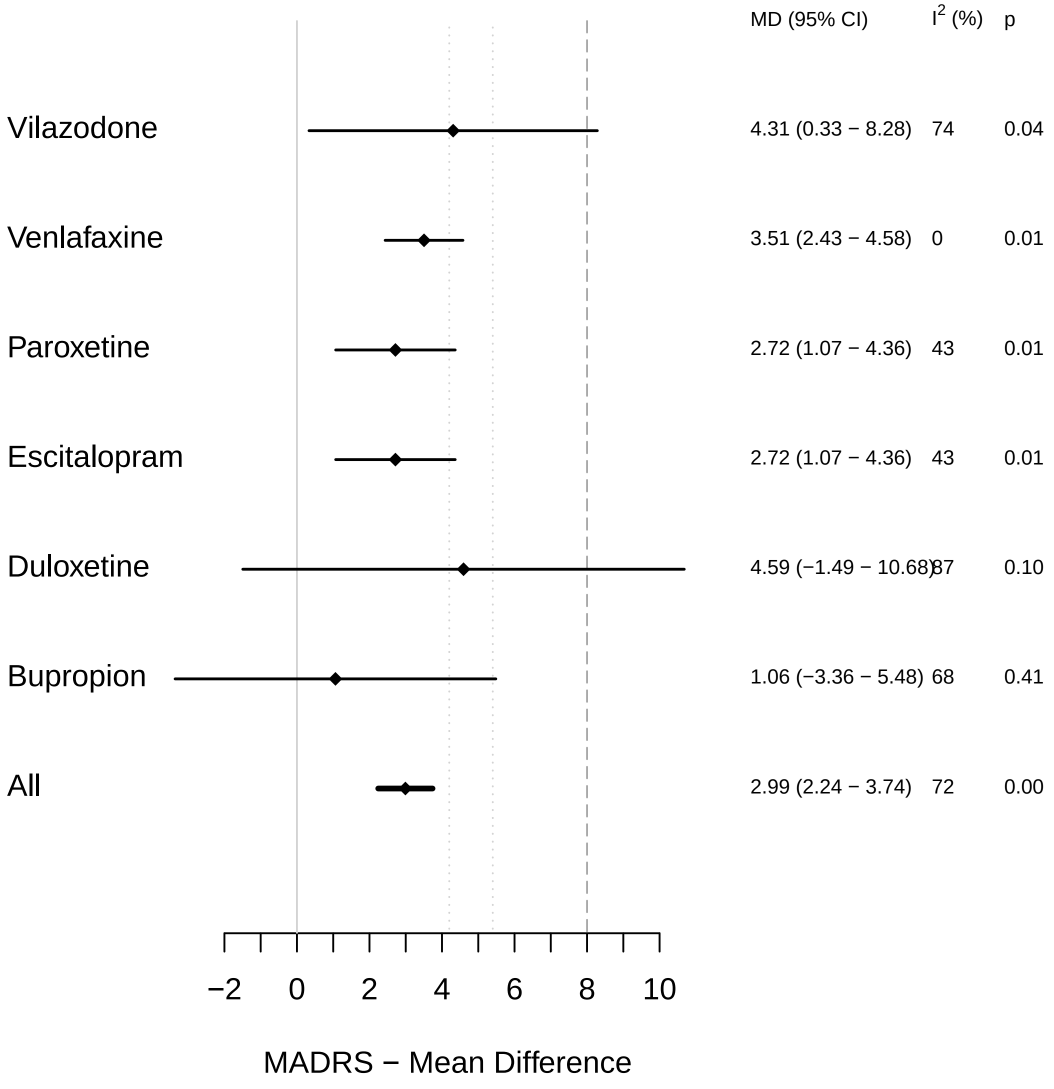

Figure 8: Meta-analytic results for trials using the MADRS and with mean differences as outcome. The vertical gray dashed line is the threshold for clinical significance according to Leucht et al. (2013), the vertical dotted lines are thresholds for minimal important differences according to Barrett et al. (2005) (see article text for details). MD: mean-difference, I^2: heterogenity index (%), p: p-value of meta-analytic result.

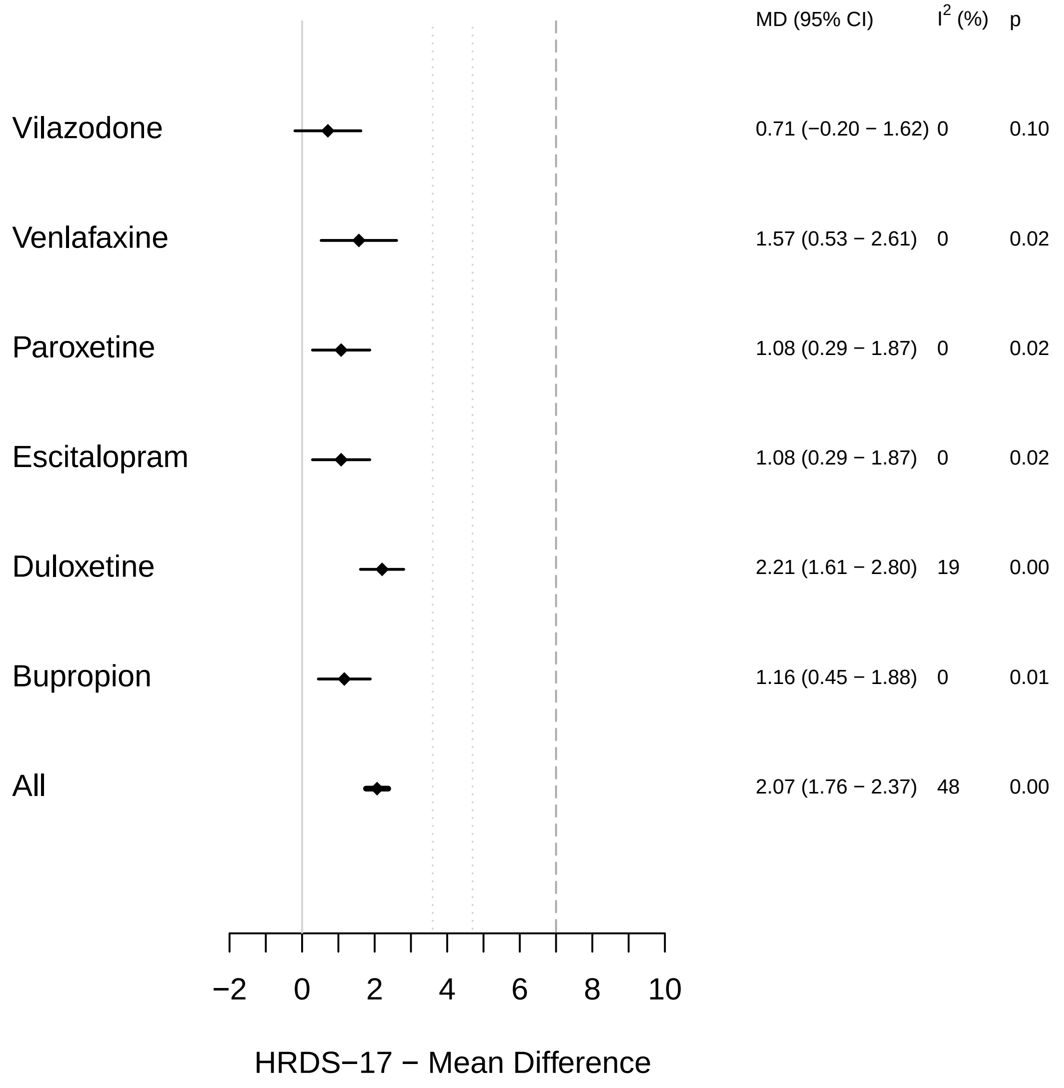

Figure 9: Meta-analytic results for trials using the HDRS-17 and with mean differences as outcome. The vertical gray dashed line is the threshold for clinical significance according to Leucht et al. (2013), the vertical dotted lines are thresholds for minimal important differences according to Barrett et al. (2005) (see article text for details). MD: mean-difference, I^2: heterogenity index (%), p: p-value of meta-analytic result.
